# Hereditary breast and ovarian cancer in Andalusian families: a genetic population study

**DOI:** 10.1186/s12885-018-4537-9

**Published:** 2018-06-08

**Authors:** Bella Pajares, Javier Porta, Jose María Porta, Cristina Fernández-de Sousa, Ignacio Moreno, Daniel Porta, Gema Durán, Tamara Vega, Inmaculada Ortiz, Carolina Muriel, Emilio Alba, Antonia Márquez

**Affiliations:** 1 Clinical Oncology Unit Hospitales Universitarios Regional y Virgen de la Victoria. Instituto de Investigación Biomédica de Málaga (IBIMA), Campus Teatinos s/n. 29010, Malaga, Spain; 2 Genologica, Paseo de la Farola 16, 29016 Malaga, Spain

**Keywords:** Hereditary breast and ovarian cancer, BRCA1/BRCA2 mutation, Genetic counselling, Recurrent mutation, Andalusian population

## Abstract

**Background:**

The BRCA1/2 mutation profile varies in Spain according to the geographical area studied. The mutational profile of BRCA1/2 in families at risk for hereditary breast and ovarian cancer has not so far been reported in Andalusia (southern Spain).

**Methods:**

We analysed BRCA1/2 germline mutations in 562 high-risk cases with breast and/or ovarian cancer from Andalusian families from 2010 to 2015.

**Results:**

Among the 562 cases, 120 (21.4%) carried a germline pathogenic mutation in BRCA1/2; 50 in BRCA1 (41.7%) and 70 in BRCA2 (58.3%). We detected 67 distinct mutations (29 in BRCA1 and 38 in BRCA2), of which 3 in BRCA1 (c.845C > A, c.1222_1223delAC, c.2527delA) and 5 in BRCA2 (c.293 T > G, c.5558_5559delGT, c.6034delT, c.6650_6654delAAGAT, c.6652delG) had not been previously described. The most frequent mutations in BRCA1 were c.5078_5080delCTG (10%) and c.5123C > A (10%), and in BRCA2 they were c.9018C > A (14%) and c.5720_5723delCTCT (8%). We identified 5 variants of unknown significance (VUS), all in BRCA2 (c.5836 T > C, c.6323G > T, c.9501 + 3A > T, c.8022_8030delGATAATGGA, c.10186A > C). We detected 76 polymorphisms (31 in BRCA1, 45 in BRCA2) not associated with breast cancer risk.

**Conclusions:**

This is the first study reporting the mutational profile of BRCA1/2 in Andalusia. We identified 21.4% of patients harbouring BRCA1/2 mutations, 58.3% of them in BRCA2. We also characterized the clinical data, mutational profile, VUS and haplotype profile.

**Electronic supplementary material:**

The online version of this article (10.1186/s12885-018-4537-9) contains supplementary material, which is available to authorized users.

## Background

**Fig. 1:**
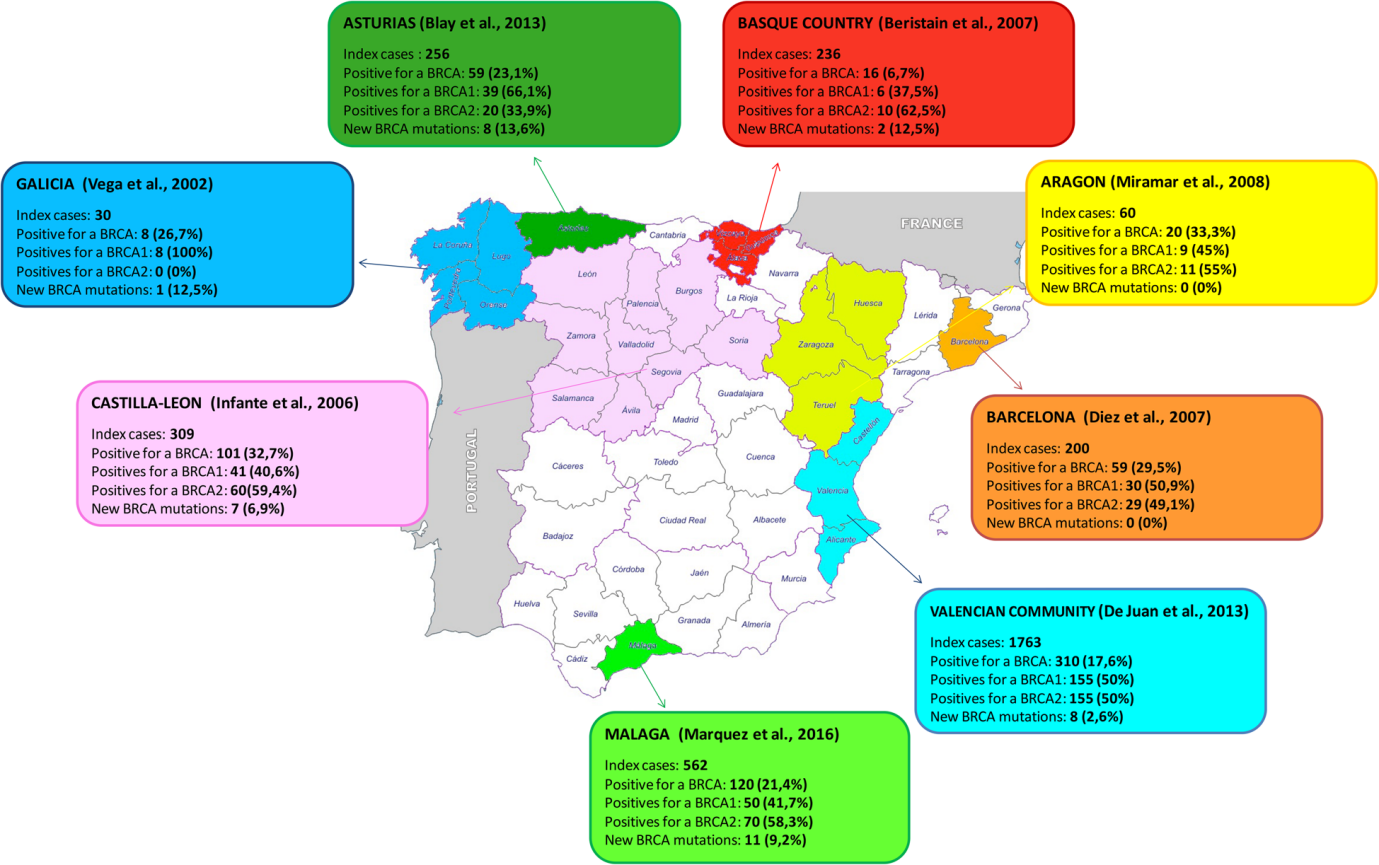
The prevalence and spectrum of BRCA1 and BRCA2 mutations in Spanish studies

About 5–10% of all breast cancer (BC) cases are due to inherited predisposition, and about 20–40% of these cases are caused by germline mutations in BRCA1 and BRCA2 genes [1, 2]. Women with BRCA1/2 germline mutations have a high lifetime risk for developing both BC and ovarian cancer (OC) compared to women from the general population [3]. Both genes have a high allelic heterogeneity and more than 3500 DNA sequence variants have been reported, including pathogenic mutations, polymorphisms and variants of unknown significance (VUS) [4]. Studies of polymorphisms and their haplotypes in BRCA1 and BRCA2 are necessary to establish the genetic structure of our population and their differences and similarities with other populations, as well as the possible relationship with the risk for BC or intrinsic subtypes. The prevalence and profile of BRCA1 and BRCA2 germline mutations show significant ethnic and geographic variation. In Spain, several studies have reported the mutational analysis of BRCA1/2 in families with hereditary breast and ovarian cancer (HBOC), noting considerable geographical variation regarding the prevalence of BRCA1 and BRCA2 pathogenic mutations, recurrent mutations, novel mutations and VUS (Fig. 1) [5]. This wide variations show that the multiple places of origin of Spanish families increases the variety of mutations in high risk HBOC spanish patients and modifies the frequency of recurrent mutations in each area. Although these studies cover many areas of Spain, none has yet been undertaken in southern Spain, the most populated region in our country and the closest to the Maghreb countries. Thus, no detailed information exists about HBOC, the mutational prevalence, the profile or polymorphisms/haplotypes of the BRCA1 and BRCA2 genes in Andalusia.

The aim of this study was to determine the mutational profile of BRCA1 and BRCA2 in 562 families at risk for HBOC from Malaga (recurrent mutations, novel mutations and VUS) and correlate the clinical characteristics of these patients with the mutational spectrum of BRCA1 and BRCA2. We also investigated genetic variants of these genes by studying BRCA1 and BRCA2 polymorphisms and haplotypes.

## Methods

### Study population

The study included a total of 562 index cases (ICs) of women at high risk for HBOC selected by the Family Cancer Unit at the Regional and the Virgen de la Victoria hospitals in Malaga, Spain, between 2010 and 2015. Families studied were unrelated, Spanish with a caucasian origin and with residence in Andalucia.

Genetic testing was offered to individuals from families at a high risk for HBOC meeting the 2011 criteria of the Spanish Society of Clinical Oncology (SEOM) [6] (Table 1). A total of 562 families fulfilled at least one of the selection criteria. The study was approved by the hospital ethics committee. All tested individuals provided signed informed consent following the appropriate genetic counselling. Genealogical trees were constructed on the basis of an index case considered to have the highest probability of being a deleterious mutation carrier (the male case or the youngest female case). None of the families met the strict criteria for other known syndromes involving BC such as Li-Fraumeni, ataxia telangiectasia or Cowden disease. Information concerning the status of oestrogen receptor (ER), progesterone receptor (PR) and HER2 protein was gathered from pathology and medical reports. The immunohistochemical technique was carried out in an automated immunostaining DAKO TechMate Horizon, using the EnVision system (DAKO) as a method for visualizing the antigen-antibody reaction. The antibodies used for staining were: Estrogen receptor (1D5, Dako); Progesterone receptor (PgR636, Dako) and c-erb-B2 (HERceptestTM, Dako). A luminal phenotype was considered when ER or PR were positive by immunohistochemistry (IHC). A triple negative phenotype was considered if the tumours were ER, PR and HER2 negative and tumours were considered HER2 positive if the HER2 protein was positive by IHC (+++) or by immunofluorescence (FISH/SISH), independently of the hormone receptor status.

**Table 1:** Selection criteria

Families with three or more second degree relatives with breast cancer or ovarian cancer, at least two of which must be first degree.	
1) Three or more family members with breast and/or ovarian cancer. (Br: Only breast cancer. Ov: at least one ovarian cancer).	
Families with two first degree relatives with breast cancer or ovarian cancer.	
2) Two family members with ovarian cancer.	
3) One family member with ovarian cancer and one with breast cancer.	
4) One family member with a male breast cancer and one with breast and/or ovarian cancer.	
5) Two family members with breast cancer before the age of 50.	
6) One family member with bilateral breast cancer and one with breast cancer, at least one before the age of 50.	
Families with a single case with breast cancer or ovarian cancer.	
7) Single affected individual with breast or ovarian cancer diagnosed before the age of 30.	
8) Single affected individual with breast and ovarian cancer.	
9) Single affected individual with bilateral breast cancer, first diagnosed before the age of 40.	

### BRCA1 and BRCA2 mutation analysis

Genomic DNA was obtained from blood using the DNeasy Blood & Tissue Kit (QiaGen) according to the manufacturer’s instructions. BRCA1 and BRCA2 coding regions and their intron–exon boundaries were amplified using PCR primers complementary to flanking intron sequences. Primers were designed by primer 3 software [7] and then evaluated by single nucleotide polymorphism (SNP) check software [8] to test for the presence of SNPs in their length, especially at the 3′ end. Sequencing reactions were performed by using an ABI Prism Big Dye Terminator v3.1 Cycle Sequencing Kit (Applied Biosystems). Sequenced PCR products were purified using CentriSeptfiltration columns (Applied Biosystems) following the manufacturer’s instructions. Sequencing was carried out using an ABI 3130 genetic analyser (Applied Biosystems). Visual inspection of base calling was used to evaluate the quality of DNA sequencing. NCBI reference sequences (RefSeq) NM_007294.3 and NM_000059.3 were used for the annotation of BRCA1 and BRCA2 variants, respectively. These RefSeq transcripts are included in the Locus Reference Genomic (LRG) data LRG_292-BRCA1 and LRG_293-BRCA2. Bi-directional sequencing review was performed using Mutation Surveyor Software (v.5.0.0, Soft Genetics, State College, PA). BRCA1/2 variant data were submitted to the Clinical Variation Database (ClinVar) [9].

### Large genomic rearrangements in BRCA1 and BRCA2

Screening for large genomic rearrangements (LGRs) in BRCA1 and BRCA2 was performed by multiplex ligation-dependent probe amplification (MLPA) using SALSA MLPA probemix P002 BRCA1 and SALSA MLPA probemix P090 BRCA2 kits according to the manufacturer’s instructions (MRC-Holland). MLPA products were analyzed using Genetic Analyzer ABI 3130 (Applied Biosystems). MLPA fragment analysis and comparative analysis were performed using Coffalyser.Net software (MRC-Holland) using 8 control samples to set up for peak height normalization and reaction quality control calculations.

### Mutation nomenclature and classification

The nomenclature of the sequence variants identified followed the guidelines of the Human Genome Variation Society (HGVS) recommendations, version 15.11 [10]. The recommendation of the American College of Medical Genetics and Genomics (ACMG) and the Association for Molecular Pathology (AMP) were followed to standardize interpretation and reporting of genomic results [11]. Five publicly accessible BRCA1 and BRCA2 variant databases were consulted for clinical classification of variants: ClinVar [9]**,** Universal Mutation Database (UMD) [12], Breast Cancer Mutation Data Base [13], Human Gene Mutation Database (HGMD) [14] and Leiden Open Variation Database (LOVD) [15], as well as the associated bibliography. In silico analysis of the VUS identified was performed using available software, such as PolyPhen 2 [16], PANTHER [17]**,** PhD-SNP [18]**,** SNAP [19], Meta-SNP [20] or SIFT [21] and four different splice-site prediction algorithms: Human Splicing Finder [22], Gene Splicer [23]**,** Splice Site Prediction [24] and MaxEntScan [25]. To predict the functional effect of indels we used PROVEAN Genome Variants software [26].

For the classification of novel mutations we have followed the criteria of the ACMG [11] (American College of Medical Genetics and Genomics). Specifically, the 8 novel mutations considered pathogenic met the following criteria: *Pathogenic very strong* criterion (PVS1) null variant (2 nonsense and 6 frameshift) in a gene where the loss of function (LOF) is a known mechanism of disease. Moderate criterion of evidence for pathogenicity (PM2); Absent from controls in Exome Sequencing Project, 1000 Genomes Project, or Exome Aggregation Consortium.

### Statistics

Statistical analysis was done using SPSS v.11 software. The estimation of mutation carrier probabilities in BRCA1 or BRCA2 genes for different individuals (tumour phenotypes, other tumours) or familial phenotypes (inclusion criteria, other tumours) was computed by conditional logistic regression with covariates (step by step; backwards Wald). Inclusion criteria were introduced as independent variables and mutation types as dependent variables. To compare two proportions we used the Z-test. The non-parametric Man-Whitney U or Kruskal-Wallis tests were used for comparison of two or more independent quantitative variables. The level of significance considered in all tests was 5%.

### Population study

Genetic variants were classified as either deleterious mutations or common genetic variants. Variants tagged as common polymorphisms were selected to make a genetic population study based on haplotype frequencies. Of all the SNPs obtained in the BRCA1 sequence during clinical testing we selected 14 SNPs (IVS4-49C/T, IVS8-58delT, Q356R, D693N, S694S, L771 L, P871L, E1038G, S1040 N, K1183R, R1347G, S1436S, S1613G, M1652I) that had previously been used to establish 10 canonical haplotypes [27] and in the BRCA2 sequence we selected the six most frequent polymorphisms (rs144848, rs1801406, rs543304, rs1799955, rs9534262, rs11571818). Haplotype pairs based on BRCA1 and BRCA2 genotypes were generated using the software DnaSP 3.00 [28]. Comparison of allele frequencies was performed using the Χ^2^ test or Fisher exact test when necessary. The strength of the association between different categories was stated using the OR and its 95% confidence intervals calculated by the exact method. The associations between BRCA1 and BRCA2 SNPs, haplotypes, BC risk, and molecular subtypes were analysed using logistic regression.

## Results

### General characterisation

A cohort of 562 index cases was analysed during 2010–2015 (following SEOM 2011 criteria. Table 1). Among the 562 index cases, 295 (52.5%) had one inclusion criterion for a high for HBOC, 157 (27.9%) had two criteria and 110 (19.6%) had three or more criteria. The most frequent criterion was “Three or more family members with breast and/or ovarian cancer” (317 cases, 56.4%), followed by “two second/first degree relatives with breast cancer under 50” (284 cases, 50.5%). The probability of harbouring BRCA mutations increased with the number of HBOC criteria. Of the 120 BRCA-mutated families, 94 (78%) met more than 1 criterion for HBOC, whereas of the 441 wild type (wt) families only 172 (39%) met more than 1 criterion for HBOC (*p* < 0.0001).

The vast majority of patients (559) had BC as the primary tumour (99.5%) and 3 had a history of OC (0.5%). These OC patients were included according to selection criteria number 2 (“two family members with ovarian cancer”). Among the BC patients, 495 were under the age of 50 (88.1%), bilateral BC was observed in 119 cases (21.2%), and 21 families (3.7%) had a male BC history. Among the 562 index cases, 356 (63.3%) had other primary tumours apart from the breast or ovarian cancer related to the syndrome studied. Colorectal cancer (CRC) was the most frequent tumour (*n* = 111, 19.8%), followed by prostate cancer (*n* = 86, 15.3%) and lung cancer (*n* = 69, 12.3%) (Additional file 1: Table S1).

Among the BRCA1-mutated families, the most frequent tumours were CRC, lung, gastric, and head and neck cancer (16, 14, 12 and 12%, respectively). Among the BRCA2-mutated families, the most frequent tumours were prostate, lung, CRC and gastric cancer (24.3, 15.7, 14.3 and 8.6%, respectively).

The presence of prostate cancer was significantly higher in BRCA2 compared to BRCA1 patients or wild type patients (*p* < 0.05). The most frequent second primary tumours according to BRCA status are shown in Additional file 2: Table S2.

Luminal was the most frequent BC tumour phenotype. The frequency of BC tumour phenotypes is shown in Additional file 3: Table S3. Among the BRCA1 patients, triple negative (TNBC) was the most frequent phenotype (44%), and among the BRCA2 patients, luminal was the most frequent phenotype (64.3%). One case of pathogenic mutation in BRCA1 and another in BRCA2 had a HER2-positive phenotype (2 and 1.4%, respectively).

### Mutational spectrum

Among the 562 index cases, 120 (21.4%) carried a germline pathogenic mutation in BRCA1/2 genes (Fig. 1, Additional file 4: Table S4 and Additional file 5: Table S5). Among the 120 positive cases, we identified 50 with BRCA1 pathogenic mutations (41.7%) and 70 with BRCA2 pathogenic mutations (58.3%)**.**

**Fig. 2:**
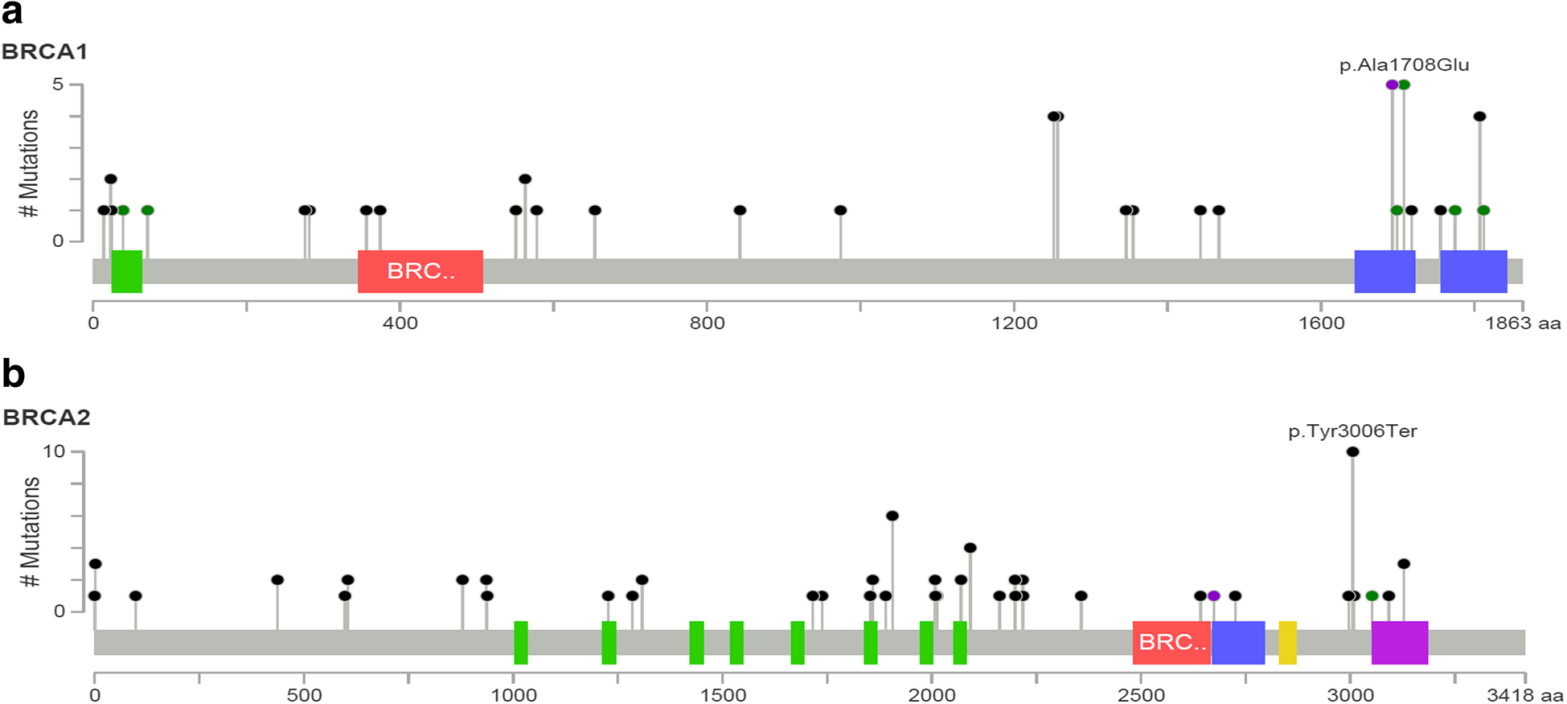
Distribution and mutational profile along BRCA 1 and BRCA2 genes Lollipop plot showing the distribution and mutation profile in **a** BRCA1 and **b** BRCA2. The truncation variants (nonsense, frameshift indels) are shown in black, missense type in green and the others in purple. The large deletions are not shown in the figure. On the vertical axis we show the frequency of appearance of each mutation. On the horizontal axis we show the aminoacid position of each mutation

Analysis of the BRCA1 gene revealed 29 distinct germline mutations. Among these 29 mutations, 3 (10.3%) are novel mutations not described so far. The most prevalent mutations were frame-shift deletions (*N* = 19; 38%), missense (*N* = 10; 20%), nonsense (*N* = 7; 14%), in-frame deletions (*N* = 5; 10%), frame-shift insertion (*N* = 4; 8%), large rearrangements (*N* = 3; 6%) and mutations in the intervening splicing sequence (*N* = 2; 4%) (Fig. 2a). We identified 3 novel frame-shift mutations that are not listed in the conventional databases (BIC, UMD, HGMD and ClinVar databases) and, as far as we know, have not been published (Table 2).

**Table 2:** Novel pathogenic mutations not described in databases

Gene	Exon	HGVS cDNA Based Designation	HGVS Protein Based Designation	Mutation Type	Criteria	Breast Cancer (BC)	BC < 50	Bilateral BC	Ovarian Cancer	Other tumours	Phenotype
BRCA1	11	c.845C > A	p.Ser282Ter	NS	1, 2, 8	YES	YES	NO	YES		Triple-negative
BRCA1	11	c.1222_1223delAC	p.Thr374Ter	FSD	1, 2, 7	YES	YES	NO	YES		Unknown
BRCA1	11	c.2527delA	p.Thr843GInfs*3	FSD	1, 5	YES	YES	NO	NO	Colon, lung	Luminal
BRCA2	7	c.293 T > G	p.Leu98Ter	NS	1, 5, 7	YES	YES	YES	NO		Luminal
BRCA2	11	c.5558_5559delGT	p.Cys1853Cysfs*4	FSD	1, 5, 7	YES	YES	YES	NO	Prostate	Luminal
BRCA2	11	c.6034delT	p.Ser2012Profs*28	FSD	1, 5	YES	YES	NO	NO		Unknown
BRCA2	11	c.6650_6654delAAGAT	p.Lys2217Ilefs*6	FSD	1, 5, 7	YES	YES	YES	YES		Luminal
BRCA2	11	c.6650_6654delAAGAT	p.Lys2217Ilefs*6	FSD	1, 3, 7	YES	YES	NO	YES	Lung	Luminal
BRCA2	11	c.6652delG	p.Asp2218Ilefs*11	FSD	1, 5, 6	YES	YES	YES	NO	Prostate	Luminal

Analysis of the BRCA2 gene revealed 38 distinct germline mutations. Among these 38 mutations, 5 (13.2%) are novel frame-shift mutations not previously described in the conventional databases (Table 2). One of the novel mutations identified in BRCA2 was present in two apparently unrelated families. The most prevalent mutations were frame-shift (*N* = 48; 68.6%), nonsense (*N* = 16; 22.9%), in-frame deletions (N = 1; 1.43%), mutations in the intervening splicing sequence (N = 4; 5.71%) and missense (N = 1; 1.43%) (Fig. 2b). We detected the same large deletion of BRCA1 exons 1–13 in three unrelated families (6%) and no deletions and/or insertions in BRCA2.

### Recurrent mutations

The 5 most recurrent mutations in BRCA1 and 5 most recurrent in BRCA2 identified in our population represent 44 and 40% of the mutations found in these genes, respectively (Tables 3 and 4). The most frequent mutations observed in BRCA1 were c.5078_5080delCTG (p.Ala1693del) and c.5123C > A (p.Ala1708Glu), followed by c.3756_3759delGTCT, c.3770_3771delAG and c.5419delA. The most recurrent BRCA2 variant in our study was c.9018C > A (p.Tyr3006Ter), identified in 10 of the 70 positive BRCA2 families (14.3%). This nonsense variant results in a premature stop codon and is predicted to encode a truncated non-functional protein. The next most recurrent BRCA2 variant, c.5720_5723delCTCT, is caused by a deletion, producing a shift in the translational reading frame and leading to a premature stop codon (p.Ser1907Terfs). No genotype-phenotype correlation was observed regarding recurrent mutations.

**Table 3:** Recurrent mutations of BRCA1 gene identified in this study

Recurrent mutations in BRCA1	Family	Criteria	Breast Cancer (BC)	Male BC	BC < 50	Bilateral BC	Ovarian Cancer	Other tumours	Phenotype
5078_5080delCTG (p.Ala1693del)	40	1, 2, 8	Yes	No	Yes	Yes	Yes	ENT	Unknown
	252	1, 5, 6	Yes	No	Yes	Yes	No	Lung, endometrial and kidney	Triple negative
	525	1, 6	Yes	No	Yes	Yes	No		Luminal
	537	2, 7, 8	Yes	No	Yes	No	Yes		Unknown
	669	1, 2, 8	Yes	No	Yes	No	Yes		Luminal
c.5123C > A (p.Ala1708Glu)	207	1, 6	Yes	No	Yes	Yes	No		Triple negative - Luminal
	507	1, 2, 8	Yes	No	Yes	No	Yes	ENT	Unknown
	811	1, 3, 5	Yes	No	Yes	No	Yes		Luminal
	836	1, 5, 6	Yes	No	Yes	Yes	No		Triple negative
	861	1, 5	Yes	No	Yes	No	No	Endometrial, gastric	Triple negative - Luminal
c.3756_3759delGTCT (p.Ser1253Argfs)	253	5	Yes	No	Yes	No	No		Unknown
	337	1, 7, 8	Yes	No	Yes	No	Yes	Lung, colon and multiple myeloma	Triple negative
	823	1, 5	Yes	No	Yes	No	No		Luminal - HER2
	1070	1, 5, 8	Yes	No	Yes	No	Yes	Cerebral and germinal tumour	Unknown
c.3770_3771delAG (p.Glu1257Glyfs)	7	1, 5	Yes	No	Yes	Yes	No	Skin	Triple negative
	62	1, 3, 6	Yes	No	Yes	No	Yes	Colon	Triple negative
	859	1, 3, 5	Yes	No	Yes	No	Yes	Colon, kidney and bladder	Triple negative
	984	1, 3, 6	Yes	No	Yes	Yes	Yes	ENT and lymphoma	Luminal
c.5419delA (p.Ile1807Leufs)	481	1, 5	Yes	No	Yes	No	No		Unknown
	690	1, 3, 5	Yes	No	Yes	No	Yes		Triple negative
	835	1, 3, 6	Yes	No	Yes	Yes	Yes	Colon and lung	Triple negative
	983	3, 5	Yes	No	Yes	No	Yes	Colon	Triple negative

**Table 4:** Recurrent mutations of BRCA2 gene identified in this study

Recurrent mutations in BRCA1	Family	Criteria	Breast Cancer (BC)	Male BC	BC < 50	Bilateral BC	Ovarian Cancer	Other tumours	Phenotype
c.9018C > A (p.Tyr3006Ter)	94	1, 6, 7	Yes	No	Yes	Yes	No	Lung	Luminal
	134	1, 2, 9	Yes	No	Yes	Yes	Yes	Melanoma	Luminal
	264	1, 5, 7	Yes	No	Yes	No	Yes	Colon and gastric	Luminal
	265	1, 5, 7	Yes	No	Yes	No	No	Lung	Luminal
	274	1, 5, 7	Yes	No	Yes	No	No	Lung	Luminal
	787	1, 6	Yes	No	Yes	Yes	No	ENT, pancreatic and lung	Luminal
	831	1	Yes	No	Yes	No	No	Colon	Triple negative
	1045	1, 6	Yes	No	Yes	Yes	No	Gastric, pancreas and prostate	Luminal
	1056	1, 2, 4	Yes	Yes	Yes	No	Yes	Colon	Luminal
	1063	5	Yes	No	Yes	No	No		Luminal
c.5720_5723delCTCT (p.Ser1907Terfs)	84	1	Yes	No	No	No	No	Thyroid	Unknown
	217	1, 4, 5	Yes	Yes	Yes	No	Yes	Lung and lymphoma	Triple negative - Luminal
	284	4, 5, 6	Yes	Yes	Yes	Yes	No	Germinal	Luminal
	286	1, 4, 5	Yes	Yes	Yes	No	No		Luminal
	824	5	Yes	No	Yes	No	No	Prostate	Luminal
	960	1, 3, 5	Yes	No	Yes	No	No	Germinal	Unknown
c.6275_6276delTT (p.Leu2092Profs)	149	1, 5	Yes	No	Yes	No	No	Prostate	Luminal
	263	1, 5	Yes	No	Yes	No	No	Prostate	Luminal
	781	1, 3, 5	Yes	No	Yes	No	Yes		Luminal
	925	5, 6	Yes	No	Yes	Yes	No	Bile duct	Luminal
c.9382C > T (p.Arg3128Ter)	77	1, 5, 6	Yes	No	Yes	Yes	No	ENT	Luminal
	291	1, 5, 7	Yes	No	Yes	No	No		Luminal
	329	1, 4, 5	Yes	Yes	Yes	Yes	No	ENT	Unknown
	?	1, 4, 5	Yes	No	Yes	No	No		Luminal
c.67 + 2 T > C	750	5, 7, 8	Yes	No	Yes	No	Yes	ENT	Luminal
	905	6	Yes	No	Yes	Yes	No	Gastric, bladder and melanoma	Luminal
	1051	5	Yes	No	Yes	No	No	Prostate, gastric and colon	Luminal

### Novel mutations

Eight new mutations were identified (not described in the BIC, UMD, HGMD or ClinVar databases). Three in BRCA1 (c.845C > A, c.1222_1223delAC, c.2527delA) and five in BRCA2 (c.293 T > G, c.5558_5559delGT, c.6034delT, c.6650_6654delAAGAT, c.6652delG) had not been previously described. Six of the new mutations identified are frameshift alterations and lead to the formation of an altered and probably non-functional protein. The other two are nonsense mutations that result in a premature stop codon. One of these novel BRCA2 mutations (p.Lys2217IlefsX6) was shared by 2 apparently unrelated families. The clinical and pathological characteristics of these families are shown in Table 2.

### Variants of unknown significance (VUS)

In our study we found 5 VUS, all of them in BRCA2 (Table 5). These VUS are described below. No VUS in coexistence with the pathogenic variants were detected in this study.

**Table 5:** BRCA2 variants of unknown significance (VUS)

Gene	c.DNA (HGVS)	Prot. (HGVS)	Variant Type	Clinical data
BRCA2	c.5836 T > C	p.Ser1946Pro	Misssense	Two cases of Breast Cancer (BC), one bilateral before 40
BRCA2	c.6323G > T	p.Arg2108Leu	Misssense	One case of BC before 30 and three cases of prostate cancer
BRCA2	c.9501 + 3A > T	IVS25 + 3A > T	Splice site	One case of Triple negative BC before 40
BRCA2	c.8022_8030delGATAATGGA	p.Lys2674Lysdel	IFD	Two cases of BC before 50
BRCA2	c.10186A > C	p.Ser3396Arg	Misssense	Two cases of BC before 50

#### BRCA2: C.5836 T > C (p.Ser1946Pro)

The variant c.5836 T > C in BRCA2 results in the change of a Serine to a Proline (p.Ser1946Pro). This variant is also defined as 6064 T > C using alternate nomenclature. The BRCA2 Ser1946Pro mutation was not observed at a significant allele frequency in 1000 genomes. Since serine and proline differ in polarity, charge, size and other properties, this is considered a non-conservative amino acid substitution. BRCA2 Ser1946Pro occurs at a position neither conserved nor located in a known functional domain. In silico analyses predict that this variant is unlikely to alter protein structure or function. Based on currently available evidence, it is unclear whether BRCA2 Ser1946Pro is a pathogenic or benign variant.

#### BRCA2: C.6323G > T (p.Arg2108Leu)

This sequence change has been reported in individuals in the Breast Cancer Information Core database [29] and the UMD [30], but has not been reported in the literature and is not present in population databases. In the UMD, this variant coexists with a pathogenic allele identified in the BRCA1 gene, which suggests that this c.6323G > T substitution in BRCA2 was not the primary cause of disease in that individual. In silico analyses predict that this variant is unlikely to alter protein structure or function but these predictions have not been confirmed by published functional studies.

#### BRCA2: C.9501 + 3A > T (IVS25 + 3A > T)

This variant consists of an A > T nucleotide substitution at the + 3 position of intron 25 of the BRCA2 gene. This variant has been observed in several breast and/or ovarian cancer families [31–33]. In vitro and in vivo RNA studies report that BRCA2 c.9501 + 3A > T results in skipping of exon 25 [33–35]. The Splicing Working Group of the Evidence-Based Network for the Interpretation of Germline Mutant Alleles (ENIGMA) concluded that BRCA2 c.9501 + 3A > T produces unequivocal splicing aberrations [36]. However, a large in vitro minigene splicing assay quantified the aberrant splicing and found that this variant results in less than 15% aberrant transcript, meaning that the full length transcript is predominant [37].

#### BRCA2: C.8022_8030delGATAATGGA (p.Lys2674Lysdel)

This variant has not been reported in the literature and is not described in variation databases (ClinVar, LOVD, InSight, UMD). This nine-nucleotide deletion in BRCA2 gives rise to a three-amino acid deletion (IME) in the putative gene product. In silico analysis by PROVEAN software predicted deleterious effects in the protein structure or function but these predictions have not been confirmed by published functional studies. This variant has been shown to co-segregate with disease in this family, but we have to consider that co-segregation of a sequence variant does not prove that the variant is causative. Based on currently available evidence, it is unclear whether BRCA2 c.8022_8030delGATAATGGA is a pathogenic or benign variant, and more studies are required to classify this variant.

#### BRCA2: C.10186A > C (p.Ser3396Arg)

This sequence change replaces serine with arginine at codon 3396 of the BRCA2 protein (p.Ser3396Arg). The serine residue is weakly conserved and there is a considerable physicochemical difference between both aminoacids. This variant is not present in population databases (ExAC without frequency) or variant databases (ClinVar, LOVD, InSight, UMD) and has not been reported in the literature in individuals with a BRCA2-related disease. Algorithms developed to predict the effect of missense changes in protein structure and function (SIFT, PolyPhen-2, Align-GVGD) suggest that this variant is tolerable, but these predictions have not been confirmed by published functional studies. In short, this is a new missense variant that is not expected to affect the function of the protein or cause disease. However, the evidence is insufficient at this time to state this conclusively.

### Polymorphisms and haplotypes

Study of genetic variants detected during clinical BRCA1 and BRCA2 mutation screening of 562 patients by direct DNA sequencing showed 76 polymorphisms (31 in BRCA1 and 45 in BRCA2).The genotype frequencies among unrelated carriers were consistent with the expected frequencies under the assumption of Hardy–Weinberg equilibrium. In general, there were no significant differences between the allele frequencies of these polymorphisms and those obtained from Exac.

**Fig. 3:**
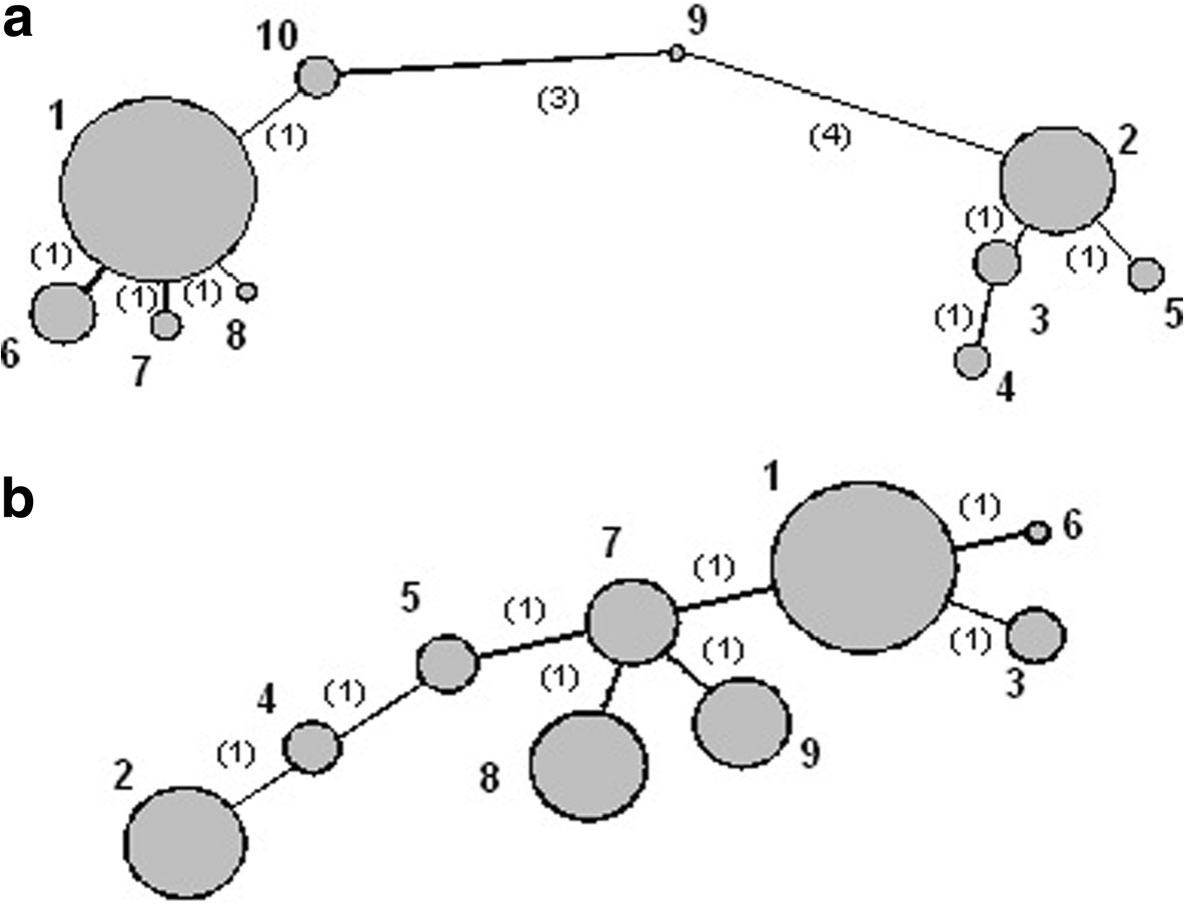
Cladogram from BRCA1 and BRCA2 haplotypes. **a** Phylogenetic tree for the ten canonical BRCA1 haplotypes. **b** Phylogenetic tree for the ten canonical BRCA2 haplotypes. Circles represent different haplotypes with the diameters being proportional to the prevalence in this study. The distance between each circle is inversely proportional to genetic relatedness between haplotypes. The numbers in parentheses indicate how many of the polymorphisms differed between haplotypes

Of the polymorphisms obtained in the BRCA1 sequence we selected 14 for the assignment of haplotype pairs to the patient samples (IVS4-49C/T, IVS8-58delT, Q356R, D693N, S694S, L771 L, P871L, E1038G, S1040 N, K1183R, R1347G, S1436S, S1613G, M1652I), as previously established by Judkins et al. [27], resulting in 10 canonical haplotypes (Fig. 3a). From the 6 polymorphisms selected in BRCA2 we obtained 9 canonical haplotypes. The results of the BRCA2 haplotype frequencies are represented by a cladogram in Fig. 3b.

No differences were found in the haplotype frequencies between BRCA mutation carriers and non carriers, nor was there any evidence of an association between the genotypes, haplotypes and BC risk. Furthermore, no significant associations emerged between individual SNPs and molecular subtypes.

## Discussion

This is the first study analysing BRCA1/BRCA2 germline mutation frequencies in Andalusia (southern Spain), the second largest autonomous community in Spain and the closest to the Maghreb. Though our results come from a single institution, the Family Cancer Unit of Malaga (Spain), they nevertheless concern a large cohort of patients (562 index cases) selected using the homogeneous criteria recommended by SEOM 2011. Regarding clinical data, the most frequent criterion in our cohort was “Three or more family members with breast and/or ovarian cancer” (317 cases, 56.4%), as observed in other Spanish studies [38–40]. We also found a high frequency of melanoma, prostate cancer and male BC in BRCA2-mutated families, concordant with published literature [41]. No male BC was detected in BRCA1-mutated families. TNBC was the most frequent phenotype in BRCA1-mutated patients and luminal in BRCA2, concordant with published studies [42, 43]. Notably, we found the unusual circumstance of one case of a pathogenic mutation in BRCA1 and another in BRCA2 presenting a HER2-positive phenotype, a molecular subtype not frequently associated with BRCA deficiency. Their characterisation by molecular features was not possible because the tumour tissue was not available.

Among the 562 index cases 120 (21.4%) carried a germline pathogenic mutation in BRCA1/2 genes. The rate of mutations in Spanish families at high risk for HBOC varies from 7 to 33% [38, 40, 44]. Our mutation rate (21.4%) is comparable to previous studies published between 2010 and 2015 with similar inclusion criteria (23%) [39, 44], but different from studies prior to 2010 (27–34%), probably due to the use of more restrictive selection criteria [39, 45, 46]. Among 120 positive cases, we identified 50 patients with BRCA1 mutations (41.7%) and 70 with BRCA2 mutations (58.3%). The prevalence and spectrum of BRCA1 and BRCA2 mutations in our population differed slightly from other Spanish studies (Fig. 1). The higher incidence of BRCA2 mutations in our study has also been reported in Extremadura [47], the Basque country [38], Castilla-León [48] and Aragon [40] but not in other regions such as Asturias [44] or Galicia [49], where the rate of BRCA1 mutations is higher than the rate of BRCA2 mutations. In Valencia BRCA1/BRCA2 ratios are similar [39]. These results are probably due to differences in the genetic background of the study population and not so much to the selection criteria or the analytical methods used.

Our study population shows clear influences from other national populations. The BRCA1 mutation c.211A > G (p.Arg71Gly), with a founding origin in Galicia and reported in 11 Spanish families [45, 50], was found once in our study. We also identified the BRCA2 frameshift mutation 5374delTATG, a highly prevalent mutation in Castilla-León (Spain). Regarding non-Spanish populations, the most common mutations in BRCA1(185delAG and 5382insC), reported in several international studies and frequent in Ashkenazi Jews (0.9 and 0.1%, respectively), was not found in our study [51, 52]. The low prevalence of both mutations in our country could be due to the absence of population mixing or large-scale migration from these areas to the Iberian Peninsula. In our study, no large rearrangements in BRCA2 were identified, which agrees with the results of others describing a higher rate of reordering in BRCA1 than in BRCA2 [53]. Regarding BRCA1, we detected the same large deletion of BRCA1 exons 1–13 in three unrelated families (6%).

Regarding recurrent mutations, the most frequent mutation observed in BRCA1 was c.5078_5080delCTG, not reported as recurrent in other national or international populations. The other more frequent recurrent BRCA1 variant present in population databases, c.5123C > A, has been reported in several Spanish studies [5, 38–40, 48, 54, 55], and is considered as recurrent in the Valencian community [39] and other geographical areas [13]. Haplotype analysis supports the idea that this mutation is a founder in Sephardic Jews and there may be a common origin in Sephardic Jews and the Spanish population [56]. The most recurrent BRCA2 variant in our study was c.9018C > A, overrepresented in our population but not frequent in other populations [45, 46, 57]. Although the recurrent mutation c.9018C > A found in ten of our families has been reported in several populations, the databases do not consider it a frequent mutation, suggesting a possible founding effect in the Andalusian territory. The second most frequent recurrent mutation was c.5720_5723delCTCT, not being described a founding origin in any population at present. To test for the presence of founder effects in c.9018C > A and c.5720_5723delCTCT, we used the genotype of the polymorphic markers linked to BRCA2. All the c.9018C > A families shared a common haplotype in 14 markers. All the c.5720_5723delCTCT families shared this common haplotype in 14 markers. Haplotype analysis supports the idea that this mutation has a founder origin in the south of Spain. We should emphasize that none of the BRCA1/2 recurrent mutations reported in North African studies (Algeria, Morocco, and Tunisia) were found in our study, despite the geographical proximity and the influence of the North African population on our ancestors [58].

An important finding of our study was the elevated number of novel mutations found (7.5%). National studies analysing the mutational profile of BRCA report novel mutation rates of 2.6–6.9%. This relatively high proportion of novel mutations can be considered a singularity of our study population that may be due to the specific characteristics and the lack of data available for southern Spain.

Concerning VUS, we have reported a list of variants identified in our study. Three of these had previously been reported while this is the first report for two of them, c.8022_8030delGATAATGGA and c.10186A > C. In recent years, many of the VUS that we found throughout this study have been reclassified through functional studies, co-segregation studies or coexistence with other pathogenic variants. However, we still do not know the importance of many variants. These variants are mostly missense, in frame deletion or possible splice site variants. This situation highlights the need to report BRCA data to databases and to publish research results. All the data obtained in this study have been reported to the Spanish Mutation Database [59] and international databases (ClinVar and LOVD).

The results of the study of polymorphisms and their derived haplotypes in BRCA1 and BRCA2 allowed us to establish the genetic structure of our population. The results of the haplotype frequencies in BRCA1 represented by a cladogram in our population were significantly similar to those obtained by Judkins in 55,630 patients from different populations. Some studies have reported associations between BRCA1 and BRCA2 SNPs and BC risk; however there is a lack of consistency across studies [60–63]. In our study, there was no evidence of an association between the genotypes, haplotypes and BC risk. Furthermore, no significant associations emerged between individual SNPs and molecular subtypes. Although study of SNP allele frequencies does not show strong differences between populations, it shows slight family differences with a possible founding effect.

Our study has some limitations such as the limited region studied (Andalusia), a single centre cohort (Malaga) and that other genes associated with a moderate-high risk for BC were not included in the analysis. On the other hand we should emphasize the large sample size (the second largest in Spain) and the homogeneous selection criteria recommended by SEOM. We should emphasize that until 2016, genetic studies regarding HBOC were practically limited to the BRCA1/BRCA2 genes, mainly due to the high cost of Sanger and MLPA sequencing techniques, but at present, most Familiar Cancer Units use gene panels related to HBOC. Since 2017 our Familiar Cancer Unit started to use a panel of 20 genes through massive sequencing that includes these and other genes related to hereditary cancer.

The BRCA1 and BRCA2 genes represent possibly the most fully sequenced genes in all human genetics. At the end of the gene-to-gene sequencing era, it is time to emphasize the importance of reporting variants and research to databases. Sharing this information is crucial for clinicians to improve patient care and allows researchers to advance in the understanding of HBOC. Currently, next-generation sequencing is providing thousands of genetic variants related to genetic diseases, and specifically HBOC. For this reason we must encourage the collection of data related to variants in BRCA1 / BRCA2 studied in the past, to investigate with more reliable information in this new era of human genetics.

## Conclusions

In conclusion, this is the first study analysing BRCA1/BRCA2 germline mutation frequencies in andalusian high risk HBOC patients. We report data from a large cohort of 562 high risk HBOC patients living in Malaga. We found 120 positive cases, 50 BRCA1- and 70 BRCA2-mutated patients. The most frequent mutations found in BRCA1 (c.5078_5080delCTG) and BRCA2 (c.9018C > A) are overrepresented in our population compared to other national and international populations. Although the recurrent mutation c.9018C > A found in ten of our families has been reported in several studies, the databases do not consider it a frequent mutation, suggesting a possible founder effect in the Andalusian territory. We also found a relatively high proportion of novel mutations (7.5%) and two VUS not reported in databases. In our study no evidence was detected of an association between the genotypes, haplotypes and BC risk and molecular subtypes.

## Additional files

Additional file 1: **Table S1.** Index cases of women at risk for HBOC and Frequency of primary tumors. (DOC 22 kb)

Additional file 2: **Table S2.** Frequency of primary tumours in BRCA mutated families. (DOC 27 kb)

Additional file 3: **Table S3.** Breast cancer tumour phenotypes according to BRCA1 and BRCA2 mutated cases. (DOC 14 kb)

Additional file 4: **Table S4.** BRCA1 pathological germline mutations according to selection criteria and clinical characteristics. (DOC 104 kb)

Additional file 5: **Table S5.** BRCA2 pathological germline mutations according to selection criteria and clinical characteristics. (DOC 140 kb)

## Abbreviations

ACMG: American College of Medical Genetics and Genomics
AMP: Association for Molecular Pathology
BC: Breast cancer
BIC: Breast Cancer Mutation Data Base
BRCT: BRCA Terminus
CRC: Colorectal cancer
ER: Oestrogen receptor
HBOC: Hereditary breast and ovary cancer
HGMD: Human Gene Mutation Database
HGVS: Human Genome Variation Society
ICs: Index cases
IHC: Immunohistochemistry
IME: Amino acid deletion
LGRSs: Large genomic rearrangements
LOVD: Leiden Open Variation Database
LRG: Locus Reference Genomic
MLPA: Multiplex ligation-dependent probe amplification
OC: Ovarian cancer
PR: Progesterone receptor
SEOM: Spanish Society of Clinical Oncology
SNP: Single nucleotide polymorphism
UMD: Universal Mutation Database
VUS: Variants of unknown significance
