# Supplementary material for: Hereditary breast and ovarian cancer in Andalusian families: a genetic population study
Source: BMC Cancer. 2018 Jun 8;18:647. doi: 10.1186/s12885-018-4537-9 (PMC5994127; doi:10.1186/s12885-018-4537-9)
Supplement: Supplementary file 1 — Table S1. Index cases of women at risk for HBOC and Frequency of primary tumors. (DOC 22 kb) [file 12885_2018_4537_MOESM1_ESM.doc]

**Table S1. Index cases of women at risk for HBOC and Frequency of primary tumors. Suppl. Mat.**

|  | **Entire cohort clinical data**  **(N: 562)** |
| --- | --- |
| **Sex** |  |
| Female | 562 (100%) |
|  |  |
| **Type of tumour** | **N (%)** |
| Breast cancer | 559 (99.5%) |
| Ovarian cancer | 135 (24%) |
| Colorectal cancer | 111 (19.8) |
| Prostate cancer | 86 (15.3) |
| Lung cancer | 69 (12.3) |
| Gastric cancer | 58 (10.3) |
| Head and neck cancer | 44 (7.8) |
| Pancreatic cancer | 28 (5) |
| Lymphoma | 28 (5) |
| Endometrial cancer | 20 (3.6) |
| Central nervous system tumours | 19 (3.4) |
| Urinary tract tumours | 15 (2.7) |
| Melanoma | 12 (2.1) |
| Thyroid cancer | 12 (2.1) |
| Hepatocarcinoma | 11 (2) |
| Germinal tumours | 10 (1.8) |
| Kidney tumours | 6 (1.1) |
| Multiple myeloma | 5 (0.9) |
| Cholangiocarcinoma | 5 (0.9) |
| Cervical cancer | 4 (0.7) |
| Osteosarcoma | 4 (0.7) |
| Unknown origin metastasis | 3 (0.5) |
| Soft tissue sarcomas | 3 (0.5) |
| Oesophagus cancer | 3 (0.5) |
| Anus tumours | 2 (0.4) |
| Penis cancer | 2 (0.4) |
| Mesothelioma | 1 (0.2) |
| Vulvar cancer | 1 (0.2) |
| Appendicular cancer | 1 (0.2) |
| Thymoma | 1 (0.2) |
|  |  |
| **Breast cancer tumour phenotypes** |  |
| Unknown | 91 (16.2%) |
| HER2 | 18 (3.2%) |
| Luminal | 360 (64.1%) |
| Triple negative | 93 (16.5%) |
