# Supplementary material for: Hereditary breast and ovarian cancer in Andalusian families: a genetic population study
Source: BMC Cancer. 2018 Jun 8;18:647. doi: 10.1186/s12885-018-4537-9 (PMC5994127; doi:10.1186/s12885-018-4537-9)
Supplement: Supplementary file 2 — Table S2. Frequency of primary tumours in BRCA mutated families. (DOC 27 kb) [file 12885_2018_4537_MOESM2_ESM.doc]

**Table S2. Frequency of primary tumours in BRCA mutated families. Suppl. Mat.**

|  | BRCA1 families | BRCA2  families |
| --- | --- | --- |
| Type of tumours | N (%) | N (%) |
| Colorectal cancer | 8 (16) | 10 (14.3) |
| Prostate cancer | 3 (6) | 17 (24.3) |
| Lung cancer | 7 (14) | 11 (15.7) |
| Gastric cancer | 6 (12) | 6 (8.6) |
| Head and neck cancer | 6 (12) | 4 (5.7) |
| Pancreatic cancer | 1 (2) | 2 (2.9) |
| Lymphoma | 2 (4) | 2 (2.9) |
| Endometrial cancer | 4 (8) | 1 (1.4) |
| Central nervous system tumours | 1 (2) | NA |
| Urinary tract tumours | 1 (2) | 4 (5.7) |
| Melanoma | NA | 4 (5.7) |
| Thyroid cancer | NA | 1 (1.4) |
| Hepatocarcinoma | NA | NA |
| Germinal tumours | 2 (4) | 3 (4.3) |
| Kidney tumours | 3 (6) | NA |
| Multiple myeloma | 1 (2) | 1 (1.4) |
| Cholangiocarcinoma | 1 (2) | 2 (2.9) |
| Cervical cancer | NA | NA |
| Osteosarcoma | 2 (4) | NA |
| Unknown origin metastasis | 1 (2) | NA |
| Soft tissue sarcomas | NA | NA |
| Oesophagus cancer | NA | 1 (1.4) |
| Anus tumours | NA | NA |
| Penis cancer | NA | NA |
| Mesothelioma | NA | NA |
| Vulvar cancer | NA | NA |
| Appendicular cancer | NA | NA |
| Thymoma | NA | NA |
