# Supplementary material for: Hereditary breast and ovarian cancer in Andalusian families: a genetic population study
Source: BMC Cancer. 2018 Jun 8;18:647. doi: 10.1186/s12885-018-4537-9 (PMC5994127; doi:10.1186/s12885-018-4537-9)
Supplement: Supplementary file 3 — Table S3. Breast cancer tumour phenotypes according to BRCA1 and BRCA2 mutated cases. (DOC 14 kb) [file 12885_2018_4537_MOESM3_ESM.doc]

**Table S3. Breast cancer tumour phenotypes according to BRCA1 and BRCA2 mutated cases. Suppl. Mat.**

|  | **BRCA1 mutated** | **BRCA2 mutated** |
| --- | --- | --- |
| **Phenotype** | **N (%)** | **N (%)** |
| Luminal | 14 (28%) | 45 (64.3%) |
| Triple negative | 22 (44%) | 8 (11.4%) |
| HER2 | 1 (2%) | 1 (1.4%) |
| Unknown | 13 (26%) | 16 (22.9%) |
|  | 50 (100%) | 70 (100%) |
