# Supplementary material for: Hereditary breast and ovarian cancer in Andalusian families: a genetic population study
Source: BMC Cancer. 2018 Jun 8;18:647. doi: 10.1186/s12885-018-4537-9 (PMC5994127; doi:10.1186/s12885-018-4537-9)
Supplement: Supplementary file 4 — Table S4. BRCA1 pathological germline mutations according to selection criteria and clinical characteristics. (DOC 104 kb) [file 12885_2018_4537_MOESM4_ESM.doc]

**Table S4. BRCA1 pathological germline mutations according to selection criteria and clinical characteristics**

| **Sample_ID** | **Gene** | **Exon** | **HGVS Protein Based Designation** | **HGVS cDNA Based Designation** | **Mutation Type** | **Criteria** | **Breast Cancer (BC)** | **Male BC** | **BC <50** | **Bilateral BC** | **Ovarian Cancer** | **Other tumours** | **Phenotype** |
| --- | --- | --- | --- | --- | --- | --- | --- | --- | --- | --- | --- | --- | --- |
| 407 | BRCA1 | 2 | p.Glu23ValfsX17 | c.68_69delAG | FSD | 5 | Yes | No | Yes | No | No | Gastric, Bile duct | Luminal |
| 501 | BRCA1 | 2 | p.Glu23ValfsX17 | c.68_69delAG | FSD | 1, 3, 7 | Yes | No | Yes | No | Yes | Osteosarcoma, | Luminal |
| 146 | BRCA1 | 2 | p.Cys24Leufs | c.70_71insTGTC | FSI | 1 | Yes | No | Yes | No | No | Lung | Triple negative |
| 806 | BRCA1 | 3 | p.Cys39Arg | c.115T>C | MS | 1, 3, 6 | Yes | No | Yes | Yes | Yes | Prostate, Gastric | Unknown |
| 106 | BRCA1 | 5 | p.Arg71Gly | c.211A>G | MS | 6 | Yes | No | Yes | Yes | No | Kidney | Triple negative |
| 584 | BRCA1 | 11 | p.Thr276AlafsX14 | c.815_824dupAGCCATGTGG | FSI | 1, 5, 8 | Yes | No | Yes | No | Yes | Gastric | Luminal |
| 303 | BRCA1 | 11 | p.Ser282Ter | c.845C>A | NS | 1, 2, 8 | Yes | No | Yes | No | Yes |  | Triple negative |
| 578 | BRCA1 | 11 | p.Gln356Ter | c.1066C>T | NS | 1, 5, 8 | Yes | No | Yes | Yes | Yes |  | Triple negative, Luminal |
| 1033 | BRCA1 | 11 | p.Thr374Ter | c.1222_1223delAC | FSD | 1, 2, 7 | Yes | No | Yes | No | Yes |  | Unknown |
| 706 | BRCA1 | 11 | p.Ser551ThrfsX4 | c.1651_1652insC | FSI | 5 | Yes | No | Yes | No | No | ENT, Endometrium | Unknown |
| 145 | BRCA1 | 11 | p.Gln563Ter | c.1687C>T | NS | 2, 3 | Yes | No | Yes | No | Yes | Lung, metastasis of unknown origin, Germinal | Luminal |
| 622 | BRCA1 | 11 | p.Gln563Ter | c.1687C>T | NS | 3 | Yes | No | No | No | Yes |  | Unknown |
| 164 | BRCA1 | 11 | p.Ser578CysfsX7 | c.1733_1734delCT | FSD | 1, 5, 6 | Yes | No | Yes | Yes | Yes | ENT | Triple negative |
| 277 | BRCA1 | 11 | p.Lys654Serfs | c.1961_1961delA | FSD | 1, 3 | Yes | No | Yes | No | Yes | Osteosarcoma | Luminal |
| 342 | BRCA1 | 11 | p.Thr843GInfs*3 | c.2527delA | FSD | 1, 5 | Yes | No | Yes | No | No | Colon, Lung | Luminal |
| 572 | BRCA1 | 11 | p.Leu974Ter | c.2921T>A | NS | 1, 5 | Yes | No | Yes | No | No | Pancreas, | HER2 |
| 253 | BRCA1 | 11 | p.Leu1252_Ser1253?fs | c.3756_3759delGTCT | FSD | 5 | Yes | No | Yes | No | No |  | Unknown |
| 823 | BRCA1 | 11 | p.Leu1252_Ser1253?fs | c.3756_3759delGTCT | FSD | 1, 5 | Yes | No | Yes | No | No |  | Luminal, HER2 |
| 1070 | BRCA1 | 11 | p.Leu1252_Ser1253?fs | c.3756_3759delGTCT | FSD | 1, 5, 8 | Yes | No | Yes | No | Yes | Brain tumor, Germinal | Unknown |
| 337 | BRCA1 | 11 | p.Leu1252_Ser1253?fs | c.3756_3759delGTCT | FSD | 1, 7, 8 | Yes | No | Yes | No | Yes | Lung, Colon, multiple myeloma | Triple negative |
| 984 | BRCA1 | 11 | p.Glu1257Glyfs | c.3770_3771delAG | FSD | 1, 3, 6 | Yes | No | Yes | Yes | Yes | ENT, Lymphoma | Luminal |
| 7 | BRCA1 | 11 | p.Glu1257Glyfs | c.3770_3771delAG | FSD | 1, 5 | Yes | No | Yes | Yes | No | Skin | Triple negative |
| 62 | BRCA1 | 11 | p.Glu1257Glyfs | c.3770_3771delAG | FSD | 1, 3, 6 | Yes | No | Yes | No | Yes | Colon | Triple negative |
| 859 | BRCA1 | 11 | p.Glu1257Glyfs | c.3770_3771delAG | FSD | 1, 3, 5 | Yes | No | Yes | No | Yes | Colon, Kidney, Bladder, | Triple negative |
| 858 | BRCA1 | 11 | p.Asn1355_Gln1356?fs | c.4065_4068delTCAA | FSD | 1, 5 | Yes | No | Yes | No | No | Prostate | Triple negative |
| 860 | BRCA1 | 13 | p.Arg1443Ter | c.4327C>T | NS | 7 | Yes | No | Yes | No | No |  | Triple negative |
| 1100 | BRCA1 | I-14 | IVS14-2A>G | c.4485-2A>G | S | 1, 3, 7 | Yes | No | Yes | No | Yes | Colon, Gastric | Triple negative, Luminal |
| 40 | BRCA1 | 18 | p.Ala1693del | c.5078_5080delCTG | IFD | 1, 2, 8 | Yes | No | Yes | Yes | Yes | ENT | Unknown |
| 252 | BRCA1 | 18 | p.Ala1693del | c.5078_5080delCTG | IFD | 1, 5, 6 | Yes | No | Yes | Yes | No | Lung, Endometrium, Kidney | Triple negative |
| 525 | BRCA1 | 18 | p.Ala1693del | c.5078_5080delCTG | IFD | 1, 6 | Yes | No | Yes | Yes | No |  | Luminal |
| 537 | BRCA1 | 18 | p.Ala1693del | c.5078_5080delCTG | IFD | 2, 7, 8 | Yes | No | Yes | No | Yes |  | Unknown |
| 669 | BRCA1 | 18 | p.Ala1693del | c.5078_5080delCTG | IFD | 1, 2, 8 | Yes | No | Yes | No | Yes |  | Luminal |
| 865 | BRCA1 | 18 | p.Arg1699Trp | c.5095C>T | MS | 1, 5, 8 | Yes | No | Yes | No | Yes | Lung, Endometrium | Luminal |
| 207 | BRCA1 | 18 | p.Ala1708Glu | c.5123C>A | MS | 1, 6 | Yes | No | Yes | Yes | No |  | Triple negative, Luminal |
| 507 | BRCA1 | 18 | p.Ala1708Glu | c.5123C>A | MS | 1, 2, 8 | Yes | No | Yes | No | Yes | ENT | Unknown |
| 811 | BRCA1 | 18 | p.Ala1708Glu | c.5123C>A | MS | 1, 3, 5 | Yes | No | Yes | No | Yes |  | Luminal |
| 861 | BRCA1 | 18 | p.Ala1708Glu | c.5123C>A | MS | 1, 5 | Yes | No | Yes | No | No | Gastric, Endometrium | Triple negative, Luminal |
| 836 | BRCA1 | 18 | p.Ala1708Glu | c.5123C>A | MS | 1, 5, 6 | Yes | No | Yes | Yes | No |  | Triple negative |
| 544 | BRCA1 | 19 | p.Trp1718Ter | c.5154G>A | NS | 5, 6, 7 | Yes | No | Yes | Yes | No |  | Triple negative |
| 954 | BRCA1 | 20 | p.Gln1756Profs*74 | c.5263_5264insC | FSI | 1, 5, 8 | Yes | No | Yes | No | Yes |  | Triple negative |
| 928 | BRCA1 | I-21 | - | c.5278-1G>T | S | 1, 5, 8 | Yes | No | Yes | No | Yes | Prostate, Bladder, ENT | Triple negative |
| 408 | BRCA1 | 21 | p.Met1775Arg | c.5324T>G | MS | 1, 5, 6 | Yes | No | Yes | Yes | Yes |  | Unknown |
| 481 | BRCA1 | 23 | p.Ile1807Leufs | c.5419delA | FSD | 1, 5 | Yes | No | Yes | No | No |  | Unknown |
| 690 | BRCA1 | 23 | p.Ile1807Leufs | c.5419delA | FSD | 1, 3, 5 | Yes | No | Yes | No | Yes |  | Triple negative |
| 835 | BRCA1 | 23 | p.Ile1807Leufs | c.5419delA | FSD | 1, 3, 6 | Yes | No | Yes | Yes | Yes | Colon, Lung | Triple negative |
| 983 | BRCA1 | 23 | p.Ile1807Leufs | c.5419delA | FSD | 3, 5 | Yes | No | Yes | No | Yes | Colon | Triple negative |
| 618 | BRCA1 | 23 | p.Pro1812Ala | c.5434C>G | MS | 1, 2 | Yes | No | No | No | Yes |  | Luminal |
| 965 | BRCA1 | Exon 1 al 13 | - | Exon1-13del | LGR | 1, 3, 7 | Yes | No | Yes | No | Yes |  | Triple negative |
| 1085 | BRCA1 | Exon 1 al 13 | - | Exon1-13del | LGR | 1, 5, 7 | Yes | No | Yes | No | No | Colon | Triple negative |
| 785 | BRCA1 | Exon 1 al 13 | - | Exon1-13del | LGR | 5 | Yes | No | Yes | No | No |  | Unknown |
