# Supplementary material for: Hereditary breast and ovarian cancer in Andalusian families: a genetic population study
Source: BMC Cancer. 2018 Jun 8;18:647. doi: 10.1186/s12885-018-4537-9 (PMC5994127; doi:10.1186/s12885-018-4537-9)
Supplement: Supplementary file 5 — Table S5. BRCA2 pathological germline mutations according to selection criteria and clinical characteristics. (DOC 140 kb) [file 12885_2018_4537_MOESM5_ESM.doc]

**Table S5. BRCA2 pathological germline mutations according to selection criteria and clinical characteristics**

| **Sample_ID** | **Gene** | **Exon** | **HGVS Protein Based Designation** | **HGVS cDNA Based Designation** | **Mutation Type** | **Criteria** | **Breast Cancer (BC)** | **Male BC** | **BC <50** | **Bilateral BC** | **Ovarian Cancer** | **Other tumours** | **Phenotype** |
| --- | --- | --- | --- | --- | --- | --- | --- | --- | --- | --- | --- | --- | --- |
| 750 | BRCA2 | 2 | IVS2+2T>C | c.67+2T>C | S | 5, 7, 8 | Yes | No | Yes | No | Yes | ENT | Luminal |
| 905 | BRCA2 | 2 | IVS2+2T>C | c.67+2T>C | S | 6 | Yes | No | Yes | Yes | No | Gastric, Bladder, Melanoma | Luminal |
| 1051 | BRCA2 | 2 | IVS2+2T>C | c.67+2T>C | S | 1, 5 | Yes | No | Yes | No | No | Prostate, Gastric, Colon | Luminal |
| 390 | BRCA2 | 7 | p.Leu98Ter | c.293T>G | NS | 1, 5, 7 | Yes | No | Yes | Yes | No |  | Luminal |
| 130 | BRCA2 | 9 |  | c.793+1G>T | S | 1, 5, 7 | Yes | No | Yes | No | Yes | Lung, Colon, Bladder | Triple negative, Luminal |
| 24 | BRCA2 | 10 | p.Lys437Argfs | c.1308_1309delGA | FSD | 1, 6 | Yes | No | Yes | Yes | No | Endometrium | Luminal |
| 1075 | BRCA2 | 10 | p.Lys437IlefsX22 | c.1310_1313delAAGA | FSD | 1, 2 | Yes | No | Yes | No | Yes |  | Luminal |
| 924 | BRCA2 | 10 | p.Thr598HisfsX16 | c.1792delA | FSD | 1, 5, 6 | Yes | No | Yes | Yes | No | Lung | Triple negative |
| 54 | BRCA2 | 10 | p.Ile605?fs | c.1813_1814insA | FSI | 1, 5, 6 | Yes | No | Yes | Yes | No | Colon | Unknown |
| 845 | BRCA2 | 10 | p.Ile605?fs | c.1813_1814insA | FSI | 5 | Yes | No | Yes | No | No |  | Luminal |
| 573 | BRCA2 | 11 | p.Ser879Terfs | c.2636_2637delCT | FSD | 1, 3 | Yes | No | Yes | No | Yes | Prostate | Luminal, HER2 |
| 985 | BRCA2 | 11 | p.Ser879Terfs | c.2636_2637delCT | FSD | 1, 6 | Yes | Yes | Yes | Yes | No | Prostate, Germinal tumour | Unknown |
| 488 | BRCA2 | 11 | p.Ala938ProfsX21 | c.2806_2809delAAAC | FSD | 1, 3 | Yes | No | Yes | No | Yes |  | Unknown |
| 22 | BRCA2 | 11 | p.Lys936_Gln937?fs | c.2808_2811delACAA | FSD | 1, 6 | Yes | No | Yes | Yes | No | Bladder | Triple negative, Luminal |
| 772 | BRCA2 | 11 | p.Lys936_Gln937?fs | c.2808_2811delACAA | FSD | 5 | Yes | No | Yes | No | No |  | Unknown |
| 850 | BRCA2 | 11 | p.Leu1227GlnfsX4 | c.3680_3681delTG | FSD | 5 | Yes | No | Yes | No | No |  | Unknown |
| 273 | BRCA2 | 11 | p.Glu1285=fs | c.3854_3854delA | FSD | 8 | Yes | No | No | No | Yes | Melanoma | Luminal |
| 29 | BRCA2 | 11 | p.Glu1308Ter | c.3922G>T | NS | 1, 5 | Yes | No | Yes | No | No | Prostate | HER2 |
| 655 | BRCA2 | 11 | p.Glu1308Ter | c.3922G>T | NS | 1, 3 | Yes | No | Yes | No | Yes | Lung | Unknown |
| 156 | BRCA2 | 11 | p.Tyr1716LysfsX8 | c.5146_5149delTATG | FSD | 1, 5 | Yes | No | Yes | No | No | Lung | Unknown |
| 660 | BRCA2 | 11 | p.Thr1738_Tyr1739?fs | c.5213_5216delCTTA | FSD | 1, 5 | Yes | No | Yes | No | No |  | Unknown |
| 402 | BRCA2 | 11 | p.Cys1853Cysfs*4 | c.5558_5559delGT | FSD | 1, 5, 7 | Yes | No | Yes | Yes | No | Prostate | Luminal |
| 283 | BRCA2 | 11 | p.Ile1859_Lys1860?fs | c.5576_5579delTTAA | FSD | 1, 3, 4 | Yes | Yes | Yes | No | Yes | Prostate | Luminal |
| 477 | BRCA2 | 11 | p.Ile1859_Lys1860?fs | c.5576_5579delTTAA | FSD | 5, 7 | Yes | No | Yes | No | No | Colon | Luminal |
| 208 | BRCA2 | 11 | p.Met1890ArgfsX8 | c.5669_5673delTGGCA | FSD | 1, 5, 8 | Yes | No | Yes | No | Yes | Melanoma | Luminal, HER2 |
| 960 | BRCA2 | 11 | p.Ser1907Terfs | c.5720_5723delCTCT | FSD | 1, 3, 5 | Yes | No | Yes | No | No | Germinal | Unknown |
| 84 | BRCA2 | 11 | p.Ser1907Terfs | c.5720_5723delCTCT | FSD | 1 | Yes | No | No | No | No | Thyroid | Unknown |
| 217 | BRCA2 | 11 | p.Ser1907Terfs | c.5720_5723delCTCT | FSD | 1, 4, 5 | Yes | Yes | Yes | No | Yes | Lung, Lymphoma, | Triple negative, Luminal |
| 284 | BRCA2 | 11 | p.Ser1907Terfs | c.5720_5723delCTCT | FSD | 4, 5, 6 | Yes | Yes | Yes | Yes | No | Germinal | Luminal |
| 286 | BRCA2 | 11 | p.Ser1907Terfs | c.5720_5723delCTCT | FSD | 1, 4, 5 | Yes | Yes | Yes | No | No |  | Luminal |
| 824 | BRCA2 | 11 | p.Ser1907Terfs | c.5720_5723delCTCT | FSD | 5 | Yes | No | Yes | No | No | Prostate | Luminal |
| 01368 | BRCA2 | 11 | p.Ser1907Terfs | c.5720_5723delCTCT | FSD | 1 | Yes | No | Yes | No | No | Pancreas | Luminal |
| 514 | BRCA2 | 11 | p.Lys2008_Gln2009?fs | c.6024_6025insG | FSI | 3, 5 | Yes | No | Yes | No | Yes |  | Luminal |
| 614 | BRCA2 | 11 | p.Lys2008_Gln2009?fs | c.6024_6025insG | FSI | 6 | Yes | No | Yes | Yes | No |  | Luminal |
| 1092 | BRCA2 | 11 | p.Gln2009Alafs | c.6024_6025insG | FSI | 6 | Yes | No | Yes | Yes | No | Prostate, Lung | Luminal |
| 52 | BRCA2 | 11 | p.Ser2012Profs*28 | c.6034delT | FSD | 1, 5 | Yes | No | Yes | No | No |  | Unknown |
| 979 | BRCA2 | 11 | p.Glu2070_Ser2071?fs | c.6209_6212delAAAG | FSD | 1, 5 | Yes | No | Yes | No | No | Prostate | Luminal |
| 506 | BRCA2 | 11 | p.Glu2070_Ser2071?fs | c.6209_6212delAAAG | FSD | 1, 5 | Yes | No | Yes | No | No | Colon, multiple myeloma | Luminal |
| 925 | BRCA2 | 11 | p.Leu2092Profs | c.6275_6276delTT | FSD | 5, 6 | Yes | No | Yes | Yes | No | Bile duct | Luminal |
| 149 | BRCA2 | 11 | p.Leu2092Profs | c.6275_6276delTT | FSD | 1, 5 | Yes | No | Yes | No | No | Prostate | Luminal |
| 263 | BRCA2 | 11 | p.Leu2092Profs | c.6275_6276delTT | FSD | 1, 5 | Yes | No | Yes | No | No | Prostate | Luminal |
| 781 | BRCA2 | 11 | p.Leu2092Profs | c.6275_6276delTT | FSD | 1, 3, 5 | Yes | No | Yes | No | Yes |  | Luminal |
| 740 | BRCA2 | 11 | p.Lys2162Asnfs | c.6486_6489delACAA | FSD | 3 | Yes | No | No | No | Yes |  | Unknown |
| 771 | BRCA2 | 11 | p.Thr2199IlefsX7 | c.6596_6596delC | FSD | 7 | Yes | No | Yes | No | No |  | Luminal |
| 841 | BRCA2 | 11 | p.Thr2199IlefsX7 | c.6596_6596delC | FSD | 5, 6 | Yes | No | Yes | Yes | No |  | Triple negative |
| 339 | BRCA2 | 11 | p.Phe2200_Ser2201PheTerfs | c.6600_6601delTT | FSD | 1 | Yes | No | Yes | No | No |  | Triple negative |
| 19 | BRCA2 | 11 | p.Lys2217Ilefs*6 | c.6650_6654delAAGAT | FSD | 1, 5, 7 | Yes | No | Yes | Yes | Yes |  | Luminal |
| 1046 | BRCA2 | 11 | p.Lys2217Ilefs*6 | c.6650_6654delAAGAT | FSD | 1, 3, 7 | Yes | No | Yes | No | Yes | Lung | Luminal |
| 90 | BRCA2 | 11 | p.Asp2218Ilefs*11 | c.6652delG | FSD | 1, 5, 6 | Yes | No | Yes | Yes | No | Prostate | Luminal |
| 592 | BRCA2 | 14 | p.Leu2357Valfs | c.7069_7070delCT | FSD | 1, 3, 5 | Yes | No | Yes | No | Yes | Prostate | Luminal |
| 124 | BRCA2 | 17 | p.Phe2642LeufsX6 | c.7926delT | FSD | 1, 6, 9 | Yes | No | Yes | Yes | No | Prostate, Colon, Gastric | Unknown |
| 784 | BRCA2 | 18 | p.Trp2725Phefs | c.8174_8185delGGTATGCTGTTAinsTT | FSD | 4 | Yes | Yes | No | Yes | No | Prostate, Bladder | Luminal |
| 672 | BRCA2 | 23 | p.Leu2996LeufsX5 | c.8988_8990delATAinsTT | FSD | 1, 4, 7 | Yes | Yes | Yes | No | No | Prostate, Gastric | Luminal |
| 94 | BRCA2 | 23 | p.Tyr3006Ter | c.9018C>A | NS | 1, 6, 7 | Yes | No | Yes | Yes | No | Lung | Luminal |
| 134 | BRCA2 | 23 | p.Tyr3006Ter | c.9018C>A | NS | 1, 2, 9 | Yes | No | Yes | Yes | Yes | Melanoma | Luminal |
| 264 | BRCA2 | 23 | p.Tyr3006Ter | c.9018C>A | NS | 1, 5, 7 | Yes | No | Yes | No | Yes | Colon, Gastric | Luminal |
| 265 | BRCA2 | 23 | p.Tyr3006Ter | c.9018C>A | NS | 1, 5, 7 | Yes | No | Yes | No | No | Lung | Luminal |
| 274 | BRCA2 | 23 | p.Tyr3006Ter | c.9018C>A | NS | 1, 5, 7 | Yes | No | Yes | No | No | Lung | Luminal |
| 1045 | BRCA2 | 23 | p.Tyr3006Ter | c.9018C>A | NS | 6 | Yes | No | Yes | Yes | No | Gastric, Prostate, Pancreas | Luminal |
| 787 | BRCA2 | 23 | p.Tyr3006Ter | c.9018C>A | NS | 1, 6 | Yes | No | Yes | Yes | No | ENT, Pancreas, Lung | Luminal |
| 831 | BRCA2 | 23 | p.Tyr3006Ter | c.9018C>A | NS | 1 | Yes | No | Yes | No | No | Colon | Triple negative |
| 1063 | BRCA2 | 23 | p.Tyr3006Ter | c.9018C>A | NS | 5 | Yes | No | Yes | No | No |  | Luminal |
| 1056 | BRCA2 | 23 | p.Tyr3006Ter | c.9018C>A | NS | 1, 2, 4 | Yes | Yes | Yes | No | Yes | Colon | Luminal |
| 30 | BRCA2 | 23 | p.Tyr3009_His3010?fs | c.9026_9030delATCAT | FSD | 1, 4, 6 | Yes | Yes | Yes | Yes | No | Oesophagus | Unknown |
| 997 | BRCA2 | 23 | p.Arg3052Trp | c.9154C>T | MS | 3 | Yes | No | No | No | Yes |  | Unknown |
| 169 | BRCA2 | 25 | p.Tyr3092llefsX12 | c.9274delT | FSD | 1 | Yes | No | Yes | No | No | Colon, Bladder, Bile duct | Luminal |
| 77 | BRCA2 | 25 | p.Arg3128Ter | c.9382C>T | NS | 1, 5, 6 | Yes | No | Yes | Yes | No | ENT | Luminal |
| 291 | BRCA2 | 25 | p.Arg3128Ter | c.9382C>T | NS | 1, 5, 7 | Yes | No | Yes | No | No |  | Luminal |
| 329 | BRCA2 | 25 | p.Arg3128Ter | c.9382C>T | NS | 1, 4, 5 | Yes | Yes | Yes | Yes | No | ENT | Unknown |
| 00329 | BRCA2 | 25 | p.Arg3128Ter | c.9382C>T | NS | 1,4, 5,6 | Yes | Yes | Yes | Yes | No | No | Unknown |
